# Bioactive and Bioadhesive Catechol Conjugated Polymers for Tissue Regeneration

**DOI:** 10.3390/polym10070768

**Published:** 2018-07-13

**Authors:** María Puertas-Bartolomé, Blanca Vázquez-Lasa, Julio San Román

**Affiliations:** 1 Institute of Polymer Science and Technology, ICTP-CSIC, Juan de la Cierva 3, 28006 Madrid, Spain; mpuertas@ictp.csic.es (M.P.-B.); jsroman@ictp.csic.es (J.S.R.); 2 CIBER’s Bioengineering, Biomaterials and Nanomedicine, CIBER-BBN, Health Institute Carlos III, C/Monforte de Lemos 3-5, Pabellón 11, 28029 Madrid, Spain

**Keywords:** wound healing, catechol, conjugated, antioxidant, anti-inflammatory, bioadhesion, ultraviolet (UV) shielding

## Abstract

The effective treatment of chronic wounds constitutes one of the most common worldwide healthcare problem due to the presence of high levels of proteases, free radicals and exudates in the wound, which constantly activate the inflammatory system, avoiding tissue regeneration. In this study, we describe a multifunctional bioactive and resorbable membrane with in-built antioxidant agent catechol for the continuous quenching of free radicals as well as to control inflammatory response, helping to promote the wound-healing process. This natural polyphenol (catechol) is the key molecule responsible for the mechanism of adhesion of mussels providing also the functionalized polymer with bioadhesion in the moist environment of the human body. To reach that goal, synthesized statistical copolymers of *N*-vinylcaprolactam (V) and 2-hydroxyethyl methacrylate (H) have been conjugated with catechol bearing hydrocaffeic acid (HCA) molecules with high yields. The system has demonstrated good biocompatibility, a sustained antioxidant response, an anti-inflammatory effect, an ultraviolet (UV) screen, and bioadhesion to porcine skin, all of these been key features in the wound-healing process. Therefore, these novel mussel-inspired materials have an enormous potential for application and can act very positively, favoring and promoting the healing effect in chronic wounds.

## 1. Introduction

To date, substantial research efforts have been directed toward developing wound-dressing materials that promote an effective treatment for skin lesions supporting the complex wound-healing process [1,2,3]. It is well known that chronic wounds, defined as wounds that do not heal (diabetic ulcers, pressure sores, venous ulcers, etc.), are extremely difficult to treat constituting one of the most common worldwide healthcare problem [4,5]. These lesions are not able to achieve functional integrity of the injured tissue after medical treatment [6], causing constant pain and diminishing the quality of life of the patient [7]. The trouble in healing chronic wounds is the continuous release of high levels of proteases, free radicals (reactive oxygen species (ROS) and reactive nitrogen species (RNOS)) as well as exudates [8,9,10,11]. Proteases degrade growth factors and elastin and collagen newly synthesized, while free radicals oxidize biomolecules and constantly activate the inflammatory system [5,7,8,12,13], and moreover, exudates promote microbial infection. These facts explain why chronic wounds remain in the inflammatory stage for too long, avoiding tissue regeneration and healing [14].

In order to promote an effective healing of chronic wounds, researchers have developed different polymeric wound dressings [15,16,17,18,19]. Since conventional wound dressings act as physical barriers and wound closure occurs as the result of the endogenous healing ability of the wound [20], new research has been currently developed based on bioactive wound dressings delivering antimicrobial agents, growth factors, antioxidant molecules etc. able to accelerate the wound-healing process [21,22,23,24]. Nevertheless, tissue toxicity has been found in wounds and surrounding areas due to the difficulties in controlling delivery the bioactive agents [25,26], and consequently, the effective control of inflammation, protease activity and free radical presence remain a great challenge [27,28,29]; hence improved approaches are still needed. In this sense, the purpose of this work lies in the development of a multifunctional bioactive and reasorbable wound dressing with in-built antioxidant agent for the continuous quenching of free radicals as well as to control inflammatory response helping to promote the healing of chronic wounds and thereby improving the efficacy of existing approaches.

Natural phenolic compounds possess important antioxidant activity [30,31,32], higher even than vitamins [33,34], and some crude plant extracts rich in phenolic groups have been used for wound healing [33,35,36,37,38]. The natural polyphenol ortho-dihydroxyphenol (catechol), has been studied in previous studies showing a great ability to assist in quenching the ROS in wounds [39,40,41,42]. In this study, catechol has been the bioactive agent chosen for the designed system in order to guarantee a persistent supply of antioxidant activity, which will continuously quench free radicals and inhibit the constant activation of the inflammatory system, promoting wound healing [43,44,45,46,47,48,49]. Catechol is the key molecule found in the byssus of mussel adhesive proteins (MAPs) secreted by the mussel’s foot and responsible for the great adherence to rocks in wet conditions [50,51]. This bioinspired kind of adhesion has been an emerging strategy developed in several researches in order to obtain wound dressings able to adhere to biological interfaces in moist environments [52,53,54,55,56,57], which is still a challenge in general surgery [58]. Thus, using catechol in the system proposed by this group, will also provide bioadhesion to the material, allowing the establishment of an intimate contact with the tissue so that the bioactive wound dressing can properly fulfill their functions. In this manner, the dressing will act as a preventive barrier for microbial infection, further physical damage and preserving a moist wound environment, which has been demonstrated to accelerate the wound re-epithelialization process [59].

Therefore, this work is focused on the preparation of resorbable and bioactive catechol conjugated polymers designed for wound-dressing purposes. The system suggested consists of conjugates of catechol with *N*-vinylcaprolactam (V) and 2-hydroxyethyl methacrylate (H) statistical copolymers, which finally bear bioactive hydrocaffeic acid (HCA) moieties. These terpolymers possess a hydrophilic character amiable with the environment of a skin lesion. Although different polymeric materials with catechol functionality have previously been reported [41,60,61,62,63], the novelty of the obtained terpolymers lies in the pathway via postpolymerization conjugation reaction to provide a flexible long-arm catechol conjugated polymer with enhanced availability of the catechol side groups. This pathway has the advantages of avoiding the drawbacks of the scavenger activity of catechol groups in polymerization reactions, protection of catechol groups is not required, while providing high yield. Thus, the developed terpolymers are directed to wound-dressing applications, in which the bioactive agent catechol will be intrinsically built into the wound dressing, providing a continuous antioxidant response, anti-inflammatory effect, and bioadhesion in the moist environment of the lesion, all of these properties being key features in the wound-healing process.

## 2. Materials and Methods

### 2.1. Materials

*N*-vinylcaprolactam (V) (Sigma-Aldrich, Saint Louis, MO, USA), 1,4-dioxane (Panreac, Barcelona, Spain), 3,4-dihydroxyhydrocinnamic acid or hydrocaffeic acid (HCA) (Sigma-Aldrich, Saint Louis, MO, USA), thionyl chloride (Scharlau, Barcelona, Spain), *N*,*N*-dimethylformamide (DMF) (Scharlau, Barcelona, Spain), toluene (Merck, Kenilworth, NJ, USA), dimethyl sulfoxide (DMSO), triethylamine (Scharlau, Barcelona, Spain), ethanol (VWR Chemicals, Radnor, PA, USA), phosphate buffered saline solution 10 mM (PBS, Arlington, TX, USA) (pH 7.4) (Sigma-Aldrich, Saint Louis, MO, USA) were used as received. 2-Hydroxyethyl methacrylate (H) (Fluka, now Sigma-Aldrich, Saint Louis, MO, USA) was previously purified according to the literature [64] and azobisisobutyronitrile (AIBN) (Fluka, now Sigma-Aldrich, Saint Louis, MO, USA) was previously crystallized in methanol (Sigma-Aldrich, Saint Louis, MO, USA).

### 2.2. Characterization Techniques

Proton nuclear magnetic resonance (^1^H-NMR) spectra were recorded at 25 °C on a Bruker Advance III HD-400 equipment (Billerica, MA, USA) in deuterated chloroform (CDCl_3_), or deuterated dimethyl sulfoxide (DMSO-*d*_6_), depending on sample. Ultraviolet (UV) spectra of the different terpolymers were recorded using a NanoDrop one (Thermo Fisher Scientific, Waltham, MA, USA). Attenuated total internal reflectance Fourier transform infrared (ATR-FTIR) spectroscopy spectra were obtained on a Perkin-Elmer (Spectrum One) spectrometer equipped with a ATR accessory. Differential scanning calorimetry (DSC) experiments were carried out on a micro-DSC-Illa apparatus (Setaram, Caluire-et-Cuire, France). Three heating-cooling cycles were examined from 25 °C to 180 °C at 10 °C/min under nitrogen at 20 mL/min flow rate. Standard Hastelloy vessels were used with 3 mg sample weight approximately. An empty vessel was used as reference. The glass transition temperature (*T*_g_) was calculated as the midpoint of the transition from the second scan. Thermogravimetric analysis (TGA) diagrams were obtained in a thermogravimetric analyzer TGA Q500 (TA instruments, New Castle, DE, USA) apparatus, under dynamic nitrogen at a heating rate of 10 °C/min in a range of 40–800 °C. From the thermograms, the temperature of 50% weight loss (*T*_50%_) and the char yield were obtained. Gel permeation chromatography (GPC) equipped with a Perkin Elmer Isocratic LC pump 250 and a refraction index detector (Series 200) was used to determine the average molecular weight and polydispersity (*M*_n_, *M*_w_ and *M*_w_/*M*_n_) of the polymers. Polystyrene-divinylbenzene columns (Waters Styragel^®^ HR, Barcelona, Spain) were used as solid phase, degassed DMF with 0.1% BrLi (0.7 mL/min) was used as eluent, and temperature was fixed at 70 °C. Monodisperse polystyrene standards (Agilent Technologies, Santa Clara, CA, USA) with molecular weights between 2930 Da and 3039 kDa were used to obtain the calibration curve. Data were analyzed using the PerkinElmer LC solution program (Waltham, MA, USA). The films’ morphology was studied using a field emission scanning electron microscope (FE-SEM), Hitachi SU-8000 (Tokyo, Japan), and atomic force microscopy (AFM) experiments (tapping mode) were carried out in a Multimode AFM (Veeco Instruments, Santa Barbara, CA, USA) with a Nanoscope IVa control system (Plainview, NY, USA, software version 6.14r1).

### 2.3. Synthesis of the VH Copolymers

*N*-vinylcaprolactam and 2-hydroxyethyl methacrylate statistical copolymers (VH) were obtained by free radical copolymerization initiated by AIBN. V and H monomers were dissolved in 1,4-dioxane with a concentration of 1 M and the solution was deoxygenated with nitrogen. Two different V:H mol % feed compositions of the monomers were used: 80:20 and 60:40. The radical initiator AIBN was carefully added to the reaction mixture with a concentration of 2.5 × 10^−2^ M and nitrogen was bubbled for 1 min. Reaction was carried out under nitrogen atmosphere at 60 °C. After 24 h, the reaction mixture was immersed in an ice bath to stop polymerization. The reaction product was dialyzed using a 3.5 kDa cut-off cellulose membrane against ethanol/water 1:1 for 48 h and against water for 48 h to remove the unreacted residues. The final product was freeze-dried, recovered and stored. Copolymer compositions were determined by NMR analysis, as described in Section 3, giving H contents of 16 and 36 mol % respectively. Hereinafter, copolymers are designated as VH16 and VH36.

### 2.4. Synthesis of the Catechol-Conjugated Polymers VHC

First, the chloride acid derivative of the hydrocaffeic acid (HCCl) was prepared according to a modified method derived from a previously reported strategy [65]: Briefty, 5 g of hydrocaffeic acid (HCA) was added to 20 mL of thionyl chloride. The mixture was stirred for 4 h under reflux (85 °C) and 10 drops of DMF were added. Then, 2 mL of toluene was added and the thionyl chloride excess was removed by distillation (80 °C) at vacuum. The HCCl was isolated as an oily orange product.

Secondly, the catechol conjugated polymers were obtained by a conjugation reaction between a fraction of the hydroxylic groups of the H units in the VH copolymers (VH16 and VH36) and an equivalent of the chloride acid derivative previously synthesized HCCl. To that end, VH copolymers were dissolved in DMF and triethylamine was added. The HCCl was dissolved in DMF/DMSO and the solution was added dropwise to the mixture. The reaction was kept for 1 day under continuous stirring and nitrogen flux at r.t. The reaction mixture was dialyzed against ethanol/water 1:1 for 1 day and against water for 2 days (cut off 3.5 kDa). The final product was freeze-dried, recovered and stored. Ultraviolet-visible (UV vis) spectroscopy was used to quantify the catechol content recording absorbance at 290 nm and comparing with standard solutions of HCA. The catechol conjugated polymers obtained from the VH16 and VH36 copolymers contained catechol fractions of 2 and 22 mol % respectively. Hereinafter, terpolymers are designated by the catechol composition values as VHC2 and VHC22.

### 2.5. Films Preparation

Thin films were obtained by a casting/solvent evaporation technique by adding 250 μL of a 25 mg/mL DMSO solution of the corresponding conjugated polymer to a glass cover (14 mm diameter) at 70 °C. Finally, films were dried until constant weight obtaining a final average thickness of 12 ± 3 μm. Films morphology was examined by FE-SEM and AFM.

### 2.6. In Vitro Degradation

The in vitro degradation of the films was examined gravimetrically under simulated physiological conditions. Briefly, the sample was initially dried and weighed (*W*_0_). Weight loss was monitored as a function of incubation time in Dulbecco’s modified Eagle’s medium (DMEM) free of serum (pH = 7.4) at 37 °C. At specific periods of time (1, 4, 7, 14 and 21 days) the samples were carefully withdrawn from the medium. Then, the samples were dried and weighed (*W*_t_). The weight loss percentage (Δ*W*%) was defined as following Equation (1):

∆*W*(%) = [(*W*_0_ − *W*_t_)/*W*_0_] × 100 (1)

### 2.7. Adhesion Strength Test

The adhesion strength of VHC polymers was examined on pig skin using a universal testing machine (UTM, Instron model 3366, Norwood, MA, USA) equipped with a 100 N load cell. The protocol of the lap shear experiment was adapted from the American Society for Testing and Materials (ASTM) standard F2255-05 (Reapproved 2015). Homogeneous test samples of fresh porcine skin with their fat removed were cut into rectangles with dimensions 40 mm length, 15 mm width and 3.5 mm thickness. Polymer and oxidation agent solutions were prepared following an adapted protocol reported in literature [66]. 50 μL of a 300 mg/mL ethanol solution of the VHC polymers in 0.01 M phosphate-buffered saline (pH 7.4) were spread on the dermis surface of one skin sample. Then, 50 μL of a 47 mM NaIO_4_ in a 10% NaOH water solution were added and mixed with the polymer solution inducing gelation. Immediately, the sample was covered with the dermis part of another piece of skin (bonding area: 15 × 10 mm^2^). Samples were covered with PBS-soaked gauze to keep the tissue moist, loaded with a normal force of 0.1 N and allowed to cure for 30 min. Adhesion strength data were collected by pulling away the two skin pieces at a rate of 5 mm/min and calculated as the maximum force divided by the overlapping adhesion area. Four replicates were tested for each composition in order to calculate the mean and standard deviation (*n* = 4). Analysis of variance (ANOVA) was performed comparing both samples at a significance level of *** *p* < 0.001 using Origin Pro 8 software (Northamptom, MA, USA) and Tukey grouping method.

### 2.8. Ultraviolet (UV) Shielding Test

An innovative method has been developed in order to evaluate the UV protective screen properties of the catechol conjugates based on the change in the wettability of porcine skin after UV irradiation. In this method, fresh porcine skin samples were cut into squares with dimensions 20 × 20 mm^2^ and the wettability was measured by analyzing the water contact angle. Subsequently, skin samples were covered with VHC terpolymer films and exposed to UV radiation generated using a UVP CL-1000 lamp with peak emission at 313 nm with an intensity of 0.95 W/m^2^. Also control skin samples were irradiated under the same conditions. Finally, the water contact angle of the skin below the terpolymer films was determined in order to compare and evaluate the UV protection of the conjugated polymers on the porcine skin. ANOVA was performed comparing the irradiated and the non-irradiated skin samples at a significance level of *** *p* < 0.001 using Origin Pro 8 software and Tukey grouping method.

### 2.9. Cellular Assays

#### 2.9.1. Cell Culture

Cellular toxicity and reactive oxygen species (ROS) assays were evaluated using human bone marrow mesenchymal stem cells (hBMSCs) (Innoprot, Derio, Vizcaya, Spain, P5), and anti-inflammatory activity was analyzed with murine RAW 264.7 macrophages (ECACC, Sigma-Aldrich, Saint Louis, MO, USA, P11). hBMSCs were cultured in DMEM enriched with 5% of fetal bovine serum (FBS), 5 mL of mesenchymal stem cell growth supplement (MSCGS), 50 μg/mL of Gentamicin (Sigma-Aldrich) and 2.5 μg/mL of Amphotericin B (Gibco, Waltham, MA, USA); and macrophages were cultured with DMEM enriched with sodium pyruvate (110 mg/L), 10% FBS, 100 μg/mL streptomycin, 100 units/mL penicillin and 200 mM l-glutamine. Cell cultures were maintained in a humidified atmosphere with 5% of CO_2_ and 95% of air. Films and cover glasses as controls were sterilized with a UV lamp (HNS Osram, 263 nm, 3.6 UVC/W) at a power of 11 W for 30 min.

#### 2.9.2. Cytotoxicity

An Alamar Blue (AB) test was used in order to indirectly analyze the cytotoxicity of the conjugated polymers. Films of both terpolymers were set up in a tube with 5 mL of FBS-free supplemented DMEM and placed on a shaker at 37 °C. Then, aliquots of medium extracts were taken at 1, 2, and 7 days under sterile conditions. hBMSCs were seeded at 9 × 10^4^ cells/mL of density and incubated to confluence for 24 h in complete medium. Then, the medium was substituted by the corresponding medium extract and incubated for 24 h. After that time, 1 mL of 10% AB in phenol red free DMEM was added and samples were incubated for 3 h. The fluorescence emission was measured (530/600 nm) on a UV multiplate reader (Biotek Synergy HT, Winooski, VT, USA) and the relative cell viability (*CV*) was calculated from Equation (2):

*CV*(%) = 100 × (*OD*_S_ − *OD*_B_)/(*OD*_C_ − *OD*_B_) (2)

where *OD*_S_, *OD*_B_ and *OD*_C_ are the optical density (*OD*) of formazan production for the sample, blank and control, respectively. Results are given as mean and standard deviation (*n* = 8).

#### 2.9.3. Reactive Oxygen Species (ROS) Quantification

Total ROS free radical activity was measured fluorometrically using 2′,7′-dichlorofluorescin diacetate (DCFH-DA) (Sigma-Aldrich). Extracts of films of both terpolymers after 24 h in PBS were taken under sterile conditions. hBMSCs were seeded at 9 × 10^4^ cells/mL of density and incubated to confluence for 24 h in complete medium. After that time the medium was removed and cells were washed three times with PBS. 200 μL of a 0.02 M DCFH-DA stock solution in PBS were added to the cells, they were incubated at 37 °C for 30 min, and washed again three times with PBS. Then, 100 μL of the samples and controls were added to each well. The positive control was a 0.02 M solution of H_2_O_2_ in PBS, the negative control was PBS and the analyzed samples consisted of 100 μL of the films’ extracts and 50/50 μL extracts/H_2_O_2_ solution. Samples were measured fluorometrically and the free radical relative content was determined by comparison. Relative fluorescence was measured at 0, 30, 60 and 120 min at 485 nm excitation/580 nm emission with a UV multiplate reader (Biotek Synergy HT, Winooski, VT, USA). Statistical analysis (ANOVA) between the different groups and the positive control at each time was performed at significance levels of * *p* < 0.05, ** *p* < 0.01 and *** *p* < 0.001 using Origin Pro 8 software and Tukey grouping method.

#### 2.9.4. Anti-inflammatory Activity

The anti-inflammatory activity of terpolymers was investigated adapting the standard protocol for nitric oxide (NO) inhibitory assay [67]. RAW 264.7 cells were seeded on the conjugated polymer films and glass covers as controls in 24-well plates at a density of 3 × 10^5^ cells/mL and they were incubated at 37 °C for 24 h. After that time, 5 μg/mL lipopolysaccharides from *E. coli* 055:B5 (LPS) were added to some of the samples and they were incubated again either with or without LPS. The nitrite concentration was determined by the Griess reaction [68,69] after 24 h, 48 h, 72 h and 1 week of incubation. The supernatant from RAW 264.7 cells were reacted with the Griess reagent (Sigma-Aldrich) (1:1) in a 96-well plate and incubated for 10 min. Production of nitrite was obtained by measuring the absorbance at 548 nm. Cellular viability (CV) of RAW 264.7 cells in the presence of the terpolymers was evaluated in parallel by using the AB assay described for cytotoxicity tests. Data were expressed as the percentage of NO production and CV, and they were given as mean ± standard deviation (*n* = 6).

## 3. Results

### 3.1. Synthesis of the VH Copolymers

**Figure 1:**
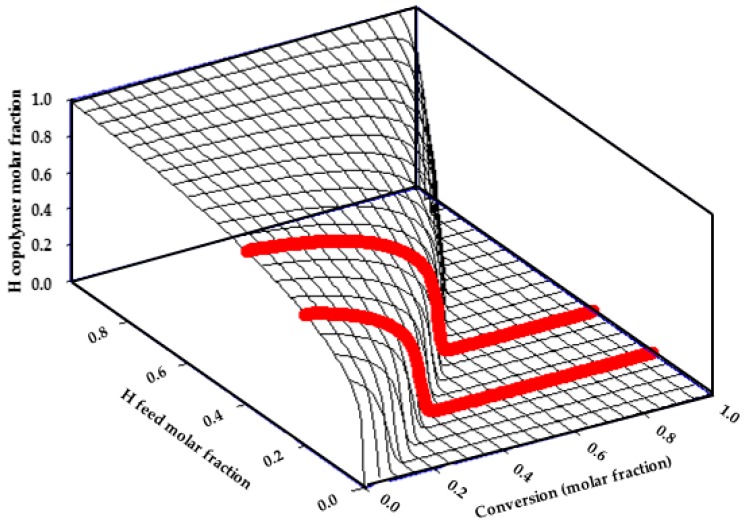
Tridimensional diagram showing the variation of instantaneous H copolymer molar fraction as a function of conversion and H feed molar fraction. Red lines represent reaction course for H feed compositions used in this work (0.2 and 0.4 mol %).

Statistical radical copolymers of V and H were synthesized at conversions of around 80% obtaining white solids in all reactions. The FTIR spectra of the copolymers are displayed in Figure S1 confirming their chemical structure, and the main bands with their corresponding assigned vibrations are described in Table S1. It can be noticed that the band corresponding to the stretching vibration of the ester group increased with the amount of H units in the copolymer while the band corresponding to the stretching vibration of the carbonyl group of the amide group decreased for the lower content of V units in the copolymer. The copolymers’ chemical structure was also determined from the ^1^H-NMR spectra. The NMR spectra and their corresponding assignments are described in Figure S2 using CDCl_3_ as solvent. Furthermore, the copolymers’ compositions (mol %) were quantitatively determined from their ^1^H-NMR spectra comparing the relative peak areas of the signal of the protons Hi of the H unit and the signal of the protons Hc of the V unit, obtaining the composition values collected in Table 1. The number average molecular weight (*M*_n_) and polydispersity (PDI = *M*_w_/*M*_n_) determined by GPC are also collected in Table 1. Reactivity ratios of these monomers, which are directly related to the comonomer distribution into the growing copolymeric chains, have been previously determined by Jansen et al. for polymerization reactions at low conversions reporting values of *r*_H_ = 7.3 and *r*_V_ = 0.01 [70]. These values indicate a much higher reactivity of the acrylic monomer (H) against the vinyl monomer (V) under the copolymerization conditions applied. Taking into consideration these reactivity values described, the kinetics of the copolymerization reaction was analyzed applying a methodology successfully employed in our research group on numerous occasions for other analogous copolymeric systems [71,72,73,74]. Results are represented in the Figure 1, where we can observe the diagram of the instantaneous H molar fraction in the copolymer chains as a function of conversion and feed molar fraction. Thick red lines represent the course of the reactions with the H feed compositions used in this work.

### 3.2. Synthesis of the Catechol Conjugated Polymers VHC

**Figure 2:**
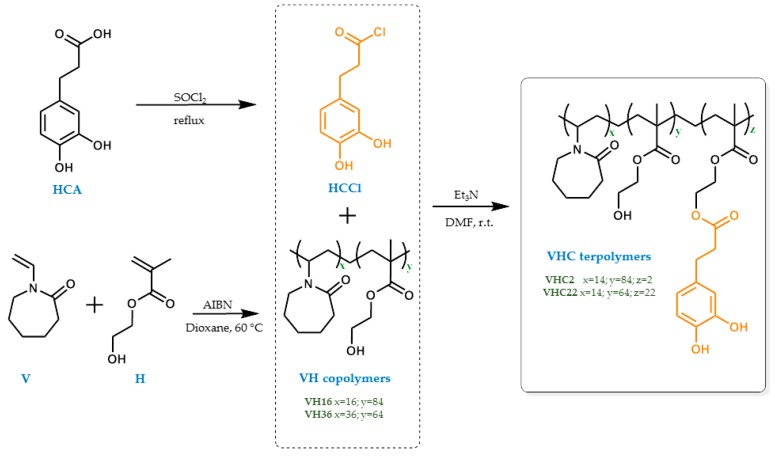
Scheme of the synthesis of the acid chloride derivative of hydrocaffeic acid (HCA), VH copolymers and the catechol conjugated polymers VHC.

The synthesis schemes of the acid chloride derivative of HCA, VH copolymers, and the catechol conjugated polymers VHC obtained after conjugation reaction are illustrated in Figure 2.

The average catechol molar composition of the terpolymers was determined by UV vis spectroscopy using a hydrocaffeic acid calibration curve at 290 nm. Taking into consideration these UV measurements, the final mol % compositions of the 3 comonomeric units compounding the terpolymers have been determined (Table 2). The chemical structure of the conjugate polymers was confirmed by FTIR spectroscopy. The FTIR spectra (Figure S3) showed the main bands belonging to the different comonomer units and their corresponding vibrations are collected in Table S2. Some differences were observed in the absorption bands of VHC polymers with respect to precursor copolymers. In particular, the band between 3200–3600 cm^−1^ became broader as a consequence of the OH bands in the catechol moieties. It can also be noticed that the band corresponding to the stretching vibration of the ester group increased with the catechol content in the terpolymers while the band corresponding to the stretching vibration of the carbonyl group of the amide group decreased and finally, the bands at 1084 and 1051 cm^−1^ increased due to the C–O stretching vibrations. NMR spectroscopy was also used to confirm the chemical structure of the conjugate polymers. The NMR spectra recorded in DMSO-*d*_6_ of the terpolymers and their corresponding proton assignments are displayed in Figure S4. Molecular weights and polidispersity of the terpolymers were measured by GPC chromatography (Table 2).

Thermal properties of the conjugated polymers were studied. TGA and differential thermogravimetric (DTGA) curves were recorded to analyze their thermal stability. VHC2 and VHC22 terpolymers showed one thermal degradation step with maxima rates at 439 °C and 432 °C respectively. The polymer VHC22 presented a higher char yield than the polymer VHC2 in nitrogen atmosphere, corresponding to its higher aromatic structure content coming from to the catechol moieties. Thermal transitions of the terpolymers were analyzed by DSC. Thermograms of VHC2 and VHC22 showed glass transition temperatures of 82 °C and 80 °C respectively, both very similar. This unique and broad transition indicates that do not exist phase segregation, which means that precursor copolymers were obtained by statistical copolymerization but, according to the diagrams in Figure 1, with a clear distribution of monomeric sequences in a gradient order. Results are presented in Table 3.

**Figure 3:**
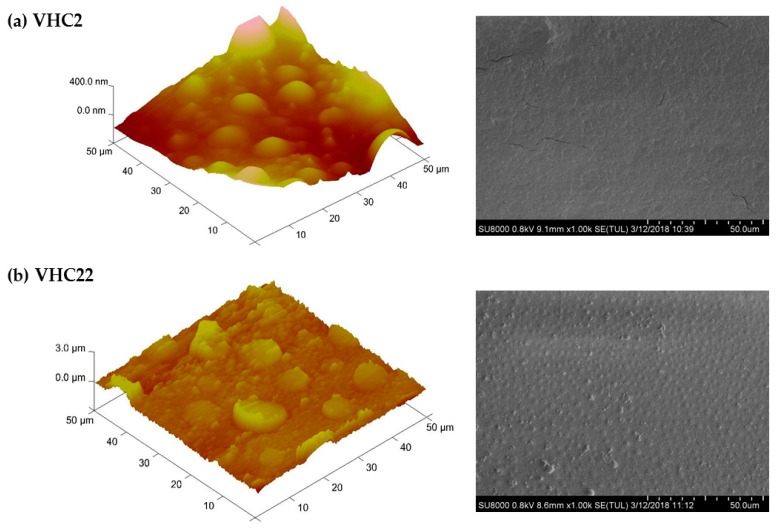
Atomic force microscopy (AFM) (**left**) and scanning electron microscopy (SEM) (**right**) images of (**a**) VHC2 terpolymer and (**b**) VHC22 terpolymer.

Surface morphology of terpolymer membranes was analyzed by SEM and AFM and images are displayed in Figure 3. Morphology of VHC2 terpolymer was rough and homogenous whereas roughness considerably increased for the system VHC22 in which, moreover, the presence of randomly distributed microdomains could be clearly observed.

### 3.3. In Vitro Degradation

**Figure 4:**
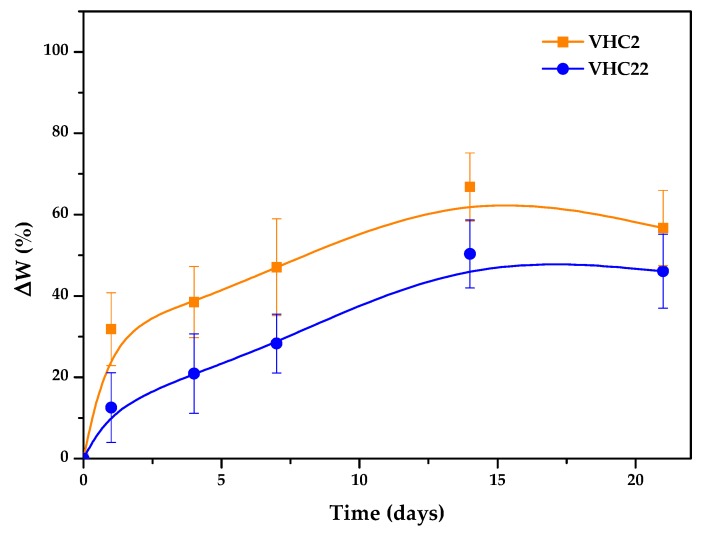
In vitro degradation kinetics of VHC films in Dulbecco’s modified Eagle’s medium (DMEM) (pH = 7.4) at 37 °C. Data are presented as mean ± standard deviation (*n* = 3).

The degradation analysis was determined gravimetrically in DMEM (pH = 7.4) at 37 °C (Figure 4). As displayed, during the incubation process an increase in weight loss of the sample with time was clearly observed. The initial degradation rate was faster in the first 24 h than in the period between 1 and 21 days, especially in the VHC2 polymer. Values of 60 and 40% weight loss were observed for VHC2 and VHC22 membranes, respectively, in the studied period. The less degradation for the VHC22 polymer in the studied period can be a consequence of the higher content of catechol groups.

### 3.4. Adhesion Strength Test

**Figure 5:**
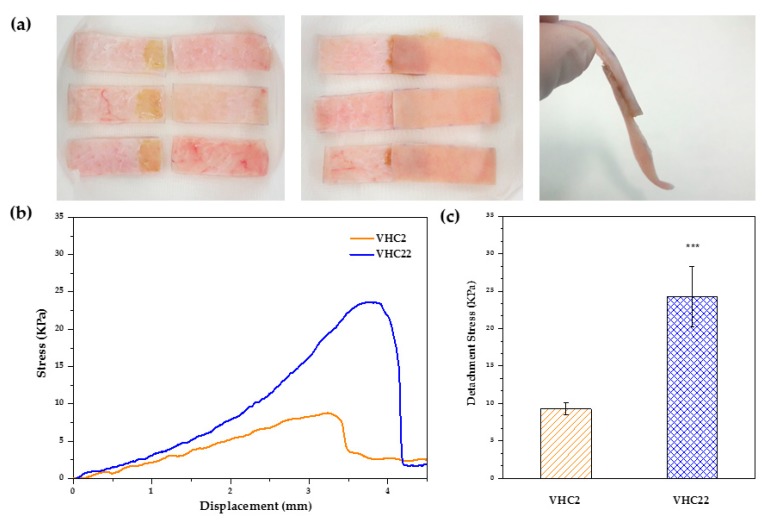
(**a**) Application of the polymer solution on the porcine tissue and skin samples attached each other. (**b**) Comparative studies in adhesion forces between the catechol conjugated polymers VHC2 and VHC22. Each line represents the stress-displacement representative curve of the two compositions after four replicates. (**c**) Detachment stress of the catechol containing polymers VHC2 and VHC22. Significant differences are denoted in the graph comparing the two groups at the significance level of *** *p* < 0.001.

Adhesion strength of VHC catechol conjugated polymers to pig skin was evaluated in a lap shear test using a UTM and following the adapted protocol of ASTM F2255-05 (Figure 5a). Figure 5b shows the (stress-displacement) curves obtained, demonstrating the higher adhesion force of the VHC22 terpolymer (22 mol % catechol) compared to the VHC2 terpolymer (2 mol % catechol). Increased detachment stress (24.3 ± 4 kPa vs. 9.3 ± 0.8 kPa) with improved ductile properties (blue curves vs. orange curves) were observed comparing the richest catechol polymer content with the lowest one. Furthermore, significant differences in the adhesive performance of both terpolymers were found as confirmed by statistical analysis using ANOVA (Figure 5c).

### 3.5. UV Shielding Test

**Figure 6:**
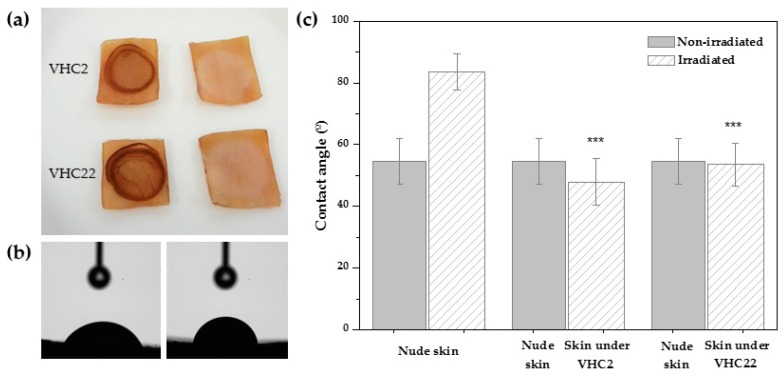
(**a**) Porcine skin samples irradiated with the terpolymer film (left) and after removing the terpolymer film (right). (**b**) Water contact angle images of the irradiated skin under de terpolymer film (left) and of the nude irradiated skin (right). (**c**) Water contact angle results of the skin control (non-irradiated and irradiated) and the skin under the VHC films. Significant differences are denoted in the graph comparing the values of the irradiated samples under the VHC films and the irradiated control skin (*** *p* < 0.001).

Water contact angles of non-irradiated skin, irradiated skin and irradiated skin covered by the VHC films were analyzed in order to study the effect of the catechol conjugates as a protective screen against UV radiation on the skin. It was observed that contact angles of the skin under the terpolymer films were similar to those of the non-irradiated skin (around 50°), characteristic of hydrophilic compounds. However, the water contact angle of the irradiated skin was much higher (around 80°), indicating that the hydrophobicity of the skin had been increased. Results are displayed in Figure 6.

### 3.6. Biological Behavior

#### 3.6.1. Cytotoxicity

**Figure 7:**
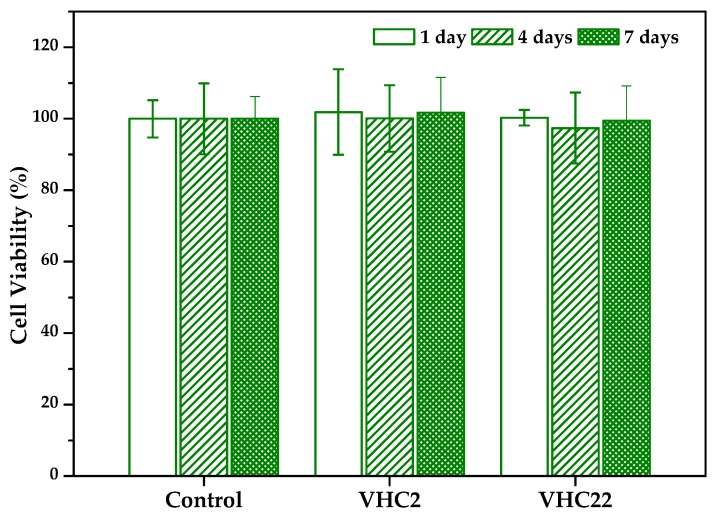
Cell viability of human bone marrow mesenchymal stem cells (hBMSCs) treated with medium extracts of VHC films taken at different times. The diagrams include the mean and the standard deviation (*n* = 8).

Indirect cytotoxicity of the conjugated polymer films at different times was analyzed by AB assay using hBMSCs. Results are shown in Figure 7. It can be observed that cell viability was not compromised with the presence of extracts of both terpolymers taken at 1, 2, and 7 days, obtaining CV values around 90–100%. This evidences the absence of in vitro cytotoxicity according to standard specifications [75].

#### 3.6.2. Antioxidant Activity

**Figure 8:**
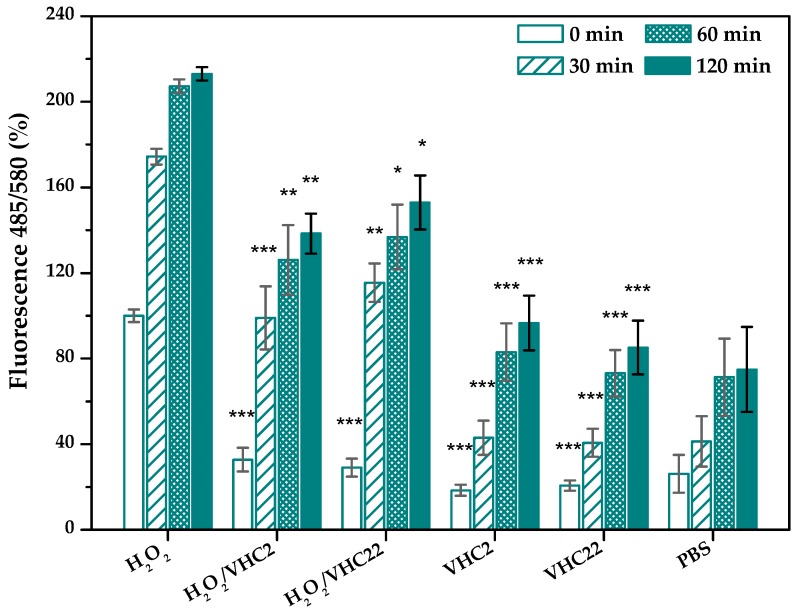
Intracellular reactive oxygen species (ROS) activity in hBMSCs measured from fluorescence emission at different times after treatment with VHC films extracts collected at 24 h. The diagrams include the mean, the standard deviation (*n* = 4) and the analysis of variance (ANOVA) between the different groups and the positive control at each time (* *p* < 0.05, ** *p* < 0.01, *** *p* < 0.001).

Both terpolymer systems efficiently reduced intracelular ROS production in vitro even when hBMSCs had been treated with H_2_O_2_ activating the oxidative reaction (Figure 8). ROS production significantly decreased with respect to H_2_O_2_ treated cells at any time, this reduction being more marked at shorter times. Furthermore, significant differences between the negative control (PBS) and VHC2 and VHC22 samples without H_2_O_2_ separately, were not found (statistical data are not included in Figure 8 for simplification purposes).

#### 3.6.3. Anti-inflammatory Activity

**Figure 9:**
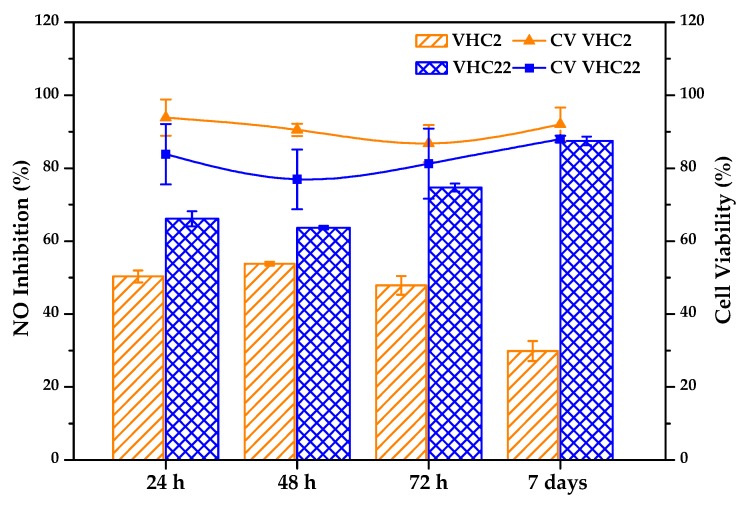
Inhibitory effects of VHC terpolymers on nitric oxide production in lipopolysaccharide (LPS) stimulated RAW 264.7 cells (bars) and cellular viability (lines and symbols).

The anti-inflammatory activity of the conjugated polymers at different times was analyzed by measuring the inhibitory effect of the polymers on the NO macrophages production. The inhibitory effects and the cell viability obtained are represented in the Figure 9. The VHC2 terpolymer showed a NO inhibition from around 50% (24 h) to 30% (1 week), considering a cell viability around 80–90%, whereas the VHC22 terpolymer presented a NO inhibitory effect from around 60% (24 h) to 90% (1 week), with a cell viability around 80–90%.

## 4. Discussion

The main purpose of this work was the preparation of bioactive membranes in order to solve the clinical demand of bioactive materials with bioadhesive properties intended for wound healing. The systems proposed consist of bioinspired films of catechol conjugated polymers. To reach that goal, firstly, statistical copolymers with a gradient distribution of monomeric sequences of V and H were synthesized at high conversions through a free radical polymerization initiated by an azo-compound using two different feed compositions. Subsequently, catechol molecule was conjugated to those copolymers by reaction between the chloride acid of HCA (previously prepared) and a portion of the hydroxyl groups of the H units in the copolymers (Figure 2). Several catechol-containing synthetic polymers have been recently developed in the family of polymethacrylates and polymethacrylamides [41,60,61,62,63]. Some of them are obtained from the synthesis of monomers containing the catechol moiety, their purification and finally their subsequent co/polymerization. However, catechol has been demonstrated to act as a chain transfer agent in the radical reaction due to the phenolic nature of the catechol group, that confers the monomer antioxidant activity acting as a radical scavenger [41,76,77,78,79]. This fact is usually related to a limited conversion, low molecular weights and a requirement of a previous protection of the catechol moieties through multiple reaction/purification steps [80,81]. Therefore, this procedure has numerous disadvantages. To solve these issues, in this work we have carried out an alternative synthetic pathway through a postpolymerization reaction on the hydroxy-functional VH copolymers, obtaining high molecular weight catechol-containing terpolymers at a relatively high yield. Polymers derived from V and H have attracted strong attention over the past years due to their biocompatible and biodegradable features, and they have been used in our group on numerous occasions [82,83,84]. In this study, V and H monomers are copolymerized to modulate the hydrophobic character of the resulting polymer. The subsequent conjugation of the catechol bioinspired molecule in the H units leads to a polymer chain with flexible and long-arm catechol side groups being obtained. The advantage of this method lies in the easier pathway via the postpolymerization conjugation reaction to prepare a high molecular weight catechol-conjugated polymer with enhanced availability of the catechol side groups promoting hydrophilic interactions with the medium. These catechol moieties provide the functionalized polymer with bioadhesive, antiinflammatory and antioxidant properties, very important features for the wound-healing process.

The behavior and chemicophysical properties of prepared catechol-conjugated polymers will be strongly dependent not only on the chemical composition of catechol but also on the dispersion of the monomeric units along the length of the polymer chains. In this sense, it is of interest to analyze the microstructure of the VH copolymeric system that will be determined by their reactivity ratios. Reactivity ratios are kinetic parameters that give information about the composition and the sequence distribution of the comonomer units along the macromolecular chains in statistical copolymers. Reactivity ratios of VH copolymers are well documented in the literature [70]. Jansen et al. obtained the reactivity ratios for reactions at low conversions employing a methodology combining RT-FTIR spectroscopy (real time-FTIR) with advanced and alternative multivariate-statistical data analysis techniques, giving values of *r*_V_ = 7.3 and *r*_H_ = 0.01. They clearly indicate the much higher reactivity of the acrylic monomer versus the vinyl monomer, which is in agreement with data found in literature where reactivity of some methacrylates are much more reactive than reactivity of V [85,86]. According to these different reactivities, the kinetics of the copolymerization will be related to the conversion degree which implies that macromolecular chains formed at low conversions contain a higher proportion of H units in the copolymer sequences, and chains formed at high conversion (after the consumption of most of H) are richer in V monomeric units. VH copolymers for the study were obtained at high conversions (around 80%), so that in this case it is interesting to analyze the tridimensional diagram of instantaneous copolymer composition variation as a function of feed composition and conversion (Figure 1), where the thick red lines correspond to the course of the reactions to obtain the synthesized copolymers VH16 and VH36. This representation was obtained using the 2004.20 algorithm “Conversion” developed in our group [87] which was successfully employed in numerous works [71,72,73,74]. In the light of this diagram, we can say that the VH copolymers are gradient polymers composed of long sequences rich in H units and long sequences rich in V units. In the case of the reactions to obtain VH16 and VH36, it can be observed that H is first consumed, as expected, and the low reactive monomer is being consumed as the reaction progresses, leading to the composition values obtained by NMR (Table 1) at high conversions (around 80%). These data give an idea of the microstructure and the composition heterogeneity of the high-conversion copolymers obtained, modulating their hydrophobic/hydrophilic balance, their solubility and stability in physiological medium, and therefore, of the catechol-conjugated polymers. After the conjugation reaction, it is expected that the terpolymers are formed by blocky sequences richer in V and blocky sequences of random HC copolymers, assuming that the conjugation reaction of catechol groups most probably is produced in a random way [88]. This microstructure will determine the chemical and physical behavior of the catechol-conjugated polymers.

The morphology of terpolymer membranes obtained by casting of the catechol conjugated polymers was observed by SEM and AFM. Images of both techniques show differences depending on the catechol content. Thus, when the terpolymer is richer in catechol groups, the presence of random microdomains distributed on the surface of the continuous matrix is evident. This behavior can be explained by the gradient microstructure of the terpolymers, their different hydrophobicity and the content of catechol-conjugated moieties (VHC2 or VHC22), all of them contributing to segregation in nano or microdomains to a greater or lesser extend depending on the content. The presence of microdomains along with the roughness of the films are two interrelated factors that will positively contribute to the adhesive properties of the system to biological tissues [89].

Bioadhesion is a very important factor for wound-healing treatments since an increase in the bioadhesion of the polymer will allow a better fit to the wound, establishing a close contact with the target tissue [52]. Furthermore, enhanced adherence to the tissue will help faster regeneration of the skin tissues [52,55,90]. Porcine skin substrate was used by its biological similarities with human dermis. Following biomimetical reasons, the skin was kept wet during the adhesion experiment in order to simulate the human damaged tissue [91]; in this way, we analyzed the effective adhesiveness of the polymers in moist environments, which is still a challenge in surgery procedures. The lap shear method, a common and reliable method for quantifying adhesion, was used [92,93]. Adhesion properties were tested at short times (30 min) in order to better observe a post-application simulation, in contrast to other reported studies that used periods in the range 12–24 h [92]. The adhesion of catechol-functionalized polymers has been recently studied on numerous occasions [66,91,93,94,95,96,97,98]. The oxidation agent NaIO_4_ has been widely used to induce crosslinking and increase bioadhesion in catechol-containing polymers [66,92,93,98]. In this work, when the NaIO_4_ solution was added to the sample deposited on the skin, the polymer solution color immediately changed to brown indicating a catechol oxidation to quinones and further cross-linking reactions. It is known that these reactive catecholquinones can react forming covalent bonds with nucleophiles found in extracellular matrix (ECM) proteins and carbohydrates of the biological tissue [99,100], improving the bioadhesion. In fact, in the tests applied in this work, we observed that both conjugated polymers failed cohesively, indicated by the brown failed polymer attached to each skin surface, demonstrating the strong interfacial adhesion force of the catechol-conjugated polymers [91]. Adhesion results evidence a higher detachment stress for the richest catechol polymer, demonstrating the key role of catechol moieties in the bioadhesion properties of the material. Furthermore, although it is difficult to directly compare the obtained results with others previously reported due to different methodologies and tissues used, it can be said that our values are in the same order of magnitude as others found in literature [66,95,101,102,103]. Overall, as far as the bioadhesive behavior is concerned, we can say that these bioinspired materials can be excellent candidates for testing in further experiments designed for application as efficient adhesives in wound-healing clinical treatments.

It is known that solar ultraviolet radiation causes various harmful effects [104,105,106,107,108], especially in damage tissue or wounds. For example, skin photo-aging is caused by UV light radiation, inducing photo-oxidative alterations such as damage and reduction in cell migration and proliferation through the production of ROS and the decrease of endogenous antioxidants of the skin [109]. Therefore, strict UV protection strategies have been currently advocated during the tissue regeneration process and wound closure [110,111,112]. Different natural compounds such as usnic acid, fern leaves, green tea, retinoids, resveratrol or *Cryptomphalus aspersa* [113,114,115] have been recently considered as potential UV-blocking sunscreens because of their antioxidant activity or their absorption on the UV region [113]. In this work, our bioinspired catechol-conjugated polymers have been studied as UV skin filters due to their UV absorption with a maximum at 290 nm and their proven antioxidant properties [39,40,41,42]. There is not a unique methodology to analyze the UV protection of materials as sunscreens, but most of the them are based on the calculation of the in vitro sun protection factor (SPF) using the erythematous effective spectrum (EES) [116], which shows that UVB rays (290–320 nm) are the most dangerous rays, with a maximum in 310 nm [117]. In this study an innovative method has been developed using porcine skin and studying the effect on the skin surface wettability, a very important aspect of the skin protective function, after being irradiated with a 310 nm UV radiation. For this purpose, water contact angle of skin samples was analyzed before and after UV irradiation. It was observed that samples covered with the terpolymer films preserved the skin surface wettability of the non-treated skin after the irradiation giving water contact values around 50°, similar to values reported in the literature [118,119]. However, for uncovered samples the skin hydrophobicity significantly increased after irradiation producing a rise in water contact angle of 30°. This fact demonstrates that the catechol absorption in the UV region is an advantageous factor assisting in antioxidant protection against UV-induced photodamage. Hence, these terpolymer systems provide a new approach for preventing UV-induced skin damage and protecting wounds from solar irradiation and they can be considered for use as a safe material whenever their biocompatibility is demonstrated.

Biocompatibility of the materials, which is closely related to cell–material interactions, is highly important in biomaterials designed for wound-healing purposes. In this work, cytotoxicity of VHC terpolymers was assessed using a hBMSCs line according to ISO 10993-5 standard which recommends testing the cell viability in the presence of extracts of samples taken at different time intervals. For that reason, in parallel, the degradation of the conjugated polymers in the same medium of extracts (DMEM free of serum) was analyzed in vitro. These experiments revealed a rather rapid degradation (measured as percentage of weight loss, ∆*W*) the extent of which depended on catechol content, being more stable the samples with higher catechol groups (∆*W* around 40% in 21 days) against the lowest catechol samples, which degraded to ∆*W* values of 60%. Nevertheless, films of both samples maintained dimensional stability. In cytotoxicity experiments, it was observed that, despite the biodegradation of the terpolymer films, cell viability values were close to 100% for all tested samples. Therefore, it can be said that the degradation of any polymer sample does not release cytotoxic rest nor at short times neither at longer times (21 days), when around 40–60% of the film has been degraded, so the cell viability of hBMSCs is not compromised during the degradation process of the catechol-conjugated polymers. Additionally, this degradation rate is enough to allow films to be degraded and replaced with regenerative tissue ingrowth.

ROS and free radicals are very important in biological systems and they have attracted increasing attention. Chronic wounds are characterized by the continuous release of proteases, ROS and high amounts of exudates [120,121]. These excessive ROS damage biomolecules and also activate the pro-inflammatory system, avoiding wound healing [14]. It is well known from the literature that phenolic acids, flavonoids etc., have excellent antioxidant properties [122]. The ability of catechol to assist in quenching the ROS in chronic wounds has already been reported [39,40]. In this way, we have carried out a cellular-based assay in order to directly evaluate the antioxidant ability of the catechol-conjugated polymers in vitro. To reach that purpose we used DCFH-DA, a non-fluorescent compound that becomes DCF (2′,7′-dichlorofluorescein) and emits fluorescence after being oxidized. By measuring the fluorescence, we were able to quantify the oxidative stress acting as a valuable indicator of oxidative stress and ROS [123,124]. Results obtained in this study indicated that both terpolymers decreased intracellular ROS production in vitro in hBMSCs previously treated with H_2_O_2_. It was also found that both conjugated polymers initially had a strong antioxidant activity which lost some effectiveness with time, independent of the catechol content, as it was observed by the authors in other low molecular catechol-containing polymers when antioxidant experiments were performed in the absence of cells [41]. Thereby, these polymers provide a source of ROS scavenger beneficial for wound-regeneration processes.

Natural polyphenolic compounds, such as catechols, have shown potent anti-inflammatory effects documented in the literature [76,78,125,126]. Anti-inflammatory activity is a crucial factor in the wound-healing process, especially in chronic wounds, which remain in the inflammatory phase preventing the healing [14,42,120]. NO inhibitory assay is a recognized experiment used to measure anti-inflammatory activity. Nitric oxide is an intermediary and regulator in pathological responses, especially in those related to acute inflammatory reactions, and LPS is a pro-inflammatory agent that activates inducible nitric oxide synthase, meaningfully increasing NO production in macrophages [67,127]. In this experiment, we have modified the method by seeding the macrophages cells directly on the polymer films. In this way, we can analyze the direct response of the cells growing on the film and being in contact with the medium released, simulating the wound-regeneration process. After LPS stimulation for 24 h, the inhibitory effects of the terpolymers on the treated macrophages were observed (Figure 8). Cell viability was also taken into consideration, eliminating the possibility that the reduction of NO is due to cytotoxicity. Terpolymers did not have a significant cytotoxicity toward macrophages cells in the presence or absence of LPS. Both terpolymers were able to decrease NO production in vitro at short times (24 h and 48 h) and this potency of suppression of NO production decreased with time for VHC2 while it increased for VHC22. These results demonstrate that anti-inflammatory activity is directly related to the catechol composition. Catechols are able to reduce NO production through two mechanisms reported in literature [126]: inhibiting the LPS signaling, and directly scavenging NO. In conclusion, this study demonstrates that these catechol-conjugated polymers have the potential to attenuate the inflammatory damage coming from the ROS generated by the cells of a wound lesion and, therefore, these systems can act very positively and can favor and promote the healing effect.

## 5. Conclusions

The synthesis of statistical VH copolymers, and the subsequent postpolymerization conjugation reaction with catechol-bearing hydrocaffeic acid (HCA) molecules, have been successfully carried out providing high molecular weight polymers with enhanced availability of the catechol side groups. These long-arm catechol moieties have been demonstrated to provide a functionalized terpolymer with bioadhesive properties to porcine skin in wet conditions; prevention for UV-induced skin damage; antioxidant properties scavenging the ROS generated by hBMSCs; and attenuation of the inflammatory damage in macrophage cultures. All of these properties are key features in the wound-healing process and, therefore, we can say that these bioinspired materials can be excellent candidates for application as efficient bioadhesive and bioactive wound dressings promoting and favoring the healing effect.

## Figures and Tables

**Table 1:** Copolymer composition values obtained from the nuclear magnetic resonance (NMR) spectra, molecular weights of the VH copolymers and reaction yields.

Copolymer	^a^*F*_H_ (mol %)	^b^*f*_H_ (mol %)	^c^*M*_n_ (Da)	^d^ PDI	Yield (%)
VH16	20	15.7	23,600	4.5	78
VH36	40	35.8	15,600	2.2	83

^a^*F* = feed composition, ^b^ *f* = copolymer composition, ^c^ *M*_n_ = number average molecular weight, ^d^ PDI = polydispersity (*M*_w_/*M*_n_).

**Table 2:** Terpolymers mol % compositions, molecular weight obtained by gel permeation chromatography (GPC) and reaction yields.

Terpolymer	*f*_V_ (mol %)	*f*_H_ (mol %)	*f*_C_ (mol %)	Yield (%)	^a^*M*_n_ (Da)	^b^ PDI
VHC2	84	13.9	2.1	13	22,200	6.7
VHC22	64	14.2	21.8	58	14,000	1.7

^a^*M*_n_ = number average molecular weight, ^b^ PDI = polydispersity (*M*_w_/*M*_n_).

**Table 3:** Thermal properties of the conjugated polymers including the maxima temperatures (*T*_max_, differential thermogravimetric (DTGA) curve), char yields and glass transition temperatures (*T*_g_).

Terpolymer	*T*_max_ (°C) main stage	Char yield (%)	*T*_g_ (°C)
VHC2	439	3.9	82
VHC22	432	8.9	80

## Supplementary Materials

FTIR and NMR spectroscopies data are available at http://www.mdpi.com/2073-4360/10/7/768/s1. Figure S1. FTIR spectra of VH copolymers. Figure S2. ^1^H-NMR spectra of VH copolymers in CDCl_3_. Figure S3. FTIR spectra of VHC terpolymers. Figure S4. ^1^H-NMR spectra of VHC terpolymers in DMSO-*d*_6_. Table S1. Wavenumber and corresponding assignments for the main peaks in FTIR spectra of VH copolymers. Table S2. Wavenumber and corresponding assignments for the main peaks in FTIR spectra of VHC terpolymers.

## References

[1] Barrientos S., Stojadinovic O., Golinko M.S., Brem H., Tomic-Canic M. (2008). Growth factors and cytokines in wound healing. Wound Repair Regen..

[2] Martin P. (1997). Wound healing—Aiming for perfect skin regeneration. Science.

[3] Rahmani Del Bakhshayesh A., Annabi N., Khalilov R., Akbarzadeh A., Samiei M., Alizadeh E., Alizadeh-Ghodsi M., Davaran S., Montaseri A. (2018). Recent advances on biomedical applications of scaffolds in wound healing and dermal tissue engineering. Artif. Cells Nanomed. Biotechnol..

[4] Braun L.R., Fisk W.A., Lev-Tov H., Kirsner R.S., Isseroff R.R. (2014). Diabetic foot ulcer: An evidence-based treatment update. Am. J. Clin. Dermatol..

[5] Brem H., Stojadinovic O., Diegelmann R.F., Entero H., Lee B., Pastar I., Golinko M., Rosenberg H., Tomic-Canic M. (2007). Molecular markers in patients with chronic wounds to guide surgical debridement. Mol. Med..

[6] Sen C.K., Gordillo G.M., Roy S., Kirsner R., Lambert L., Hunt T.K., Gottrup F., Gurtner G.C., Longaker M.T. (2009). Human skin wounds: A major and snowballing threat to public health and the economy. Wound Repair Regen..

[7] Gibson D.J., Schultz G. (2009). Chronic wound diagnostic for matrix metalloproteinase: Chronic wounds. Wound Heal. S. Afr..

[8] Wiegand C., Hipler U.C. (2010). Polymer-Based Biomaterials as Dressings for Chronic Stagnating Wounds.

[9] Bjarnsholt T., Kirketerp-Møller K., Jensen P.Ø., Madsen K.G., Phipps R., Krogfelt K., Høiby N., Givskov M. (2008). Why chronic wounds will not heal: A novel hypothesis. Wound Repair Regen..

[10] Novo E., Parola M. (2008). Redox mechanisms in hepatic chronic wound healing and fibrogenesis. Fibrogenes. Tissue Repair.

[11] Eming S.A., Krieg T., Davidson J.M. (2007). Inflammation in wound repair: Molecular and cellular mechanisms. J. Investig. Dermatol..

[12] Rojkind M., Dominguez-Rosales J.-A., Nieto N., Greenwel P. (2002). Role of hydrogen peroxide and oxidative stress in healing responses. Cell. Mol. Life Sci. CMLS.

[13] Cawston T.E., Wilson A.J. (2006). Understanding the role of tissue degrading enzymes and their inhibitors in development and disease. Best Pract. Res. Clin. Rheumatol..

[14] Nyanhongo G.S., Sygmund C., Ludwig R., Prasetyo E.N., Guebitz G.M. (2013). Synthesis of multifunctional bioresponsive polymers for the management of chronic wounds. J. Biomed. Mater. Res. Part B Appl. Biomater..

[15] Li X., Chen S., Zhang B., Li M., Diao K., Zhang Z., Li J., Xu Y., Wang X., Chen H. (2012). In situ injectable nano-composite hydrogel composed of curcumin, *N*,*O*-carboxymethyl chitosan and oxidized alginate for wound healing application. Int. J. Pharm..

[16] Li X., Nan K., Li L., Zhang Z., Chen H. (2012). In vivo evaluation of curcumin nanoformulation loaded methoxy poly(ethylene glycol)-graft-chitosan composite film for wound healing application. Carbohydr. Polym..

[17] Khil M.S., Cha D.I., Kim H.Y., Kim I.S., Bhattarai N. (2003). Electrospun nanofibrous polyurethane membrane as wound dressing. J. Biomed. Mater. Res. Part B Appl. Biomater..

[18] Schwartz V.B., Thétiot F., Ritz S., Pütz S., Choritz L., Lappas A., Förch R., Landfester K., Jonas U. (2012). Antibacterial surface coatings from zinc oxide nanoparticles embedded in poly(*N*-isopropylacrylamide) hydrogel surface layers. Adv. Funct. Mater..

[19] GhavamiNejad A., Park C.H., Kim C.S. (2016). In situ synthesis of antimicrobial silver nanoparticles within antifouling zwitterionic hydrogels by catecholic redox chemistry for wound healing application. Biomacromolecules.

[20] Sweitzer S.M., Fann S.A., Borg T.K., Baynes J.W., Yost M.J. (2006). What is the future of diabetic wound care?. Diabetes Educ..

[21] Werner S., Grose R. (2003). Regulation of wound healing by growth factors and cytokines. Physiol. Rev..

[22] Dworniczek E., Nawrot U., Seniuk A., Wlodarczyk K., Bialynicki-Birula R. (2009). The in vitro effect of a silver-containing dressing on biofilm development. Adv. Clin. Exp. Med..

[23] Jain R., Agarwal A., Kierski P.R., Schurr M.J., Murphy C.J., McAnulty J.F., Abbott N.L. (2013). The use of native chemical functional groups presented by wound beds for the covalent attachment of polymeric microcarriers of bioactive factors. Biomaterials.

[24] Huang X., Bao X., Wang Z., Hu Q. (2017). A novel silver-loaded chitosan composite sponge with sustained silver release as a long-lasting antimicrobial dressing. RSC Adv..

[25] Atiyeh B.S., Costagliola M., Hayek S.N., Dibo S.A. (2007). Effect of silver on burn wound infection control and healing: Review of the literature. Burns.

[26] Dunn K., Edwards-Jones V. (2004). The role of acticoat™ with nanocrystalline silver in the management of burns. Burns.

[27] Boateng J.S., Matthews K.H., Stevens H.N., Eccleston G.M. (2008). Wound healing dressings and drug delivery systems: A review. J. Pharm. Sci..

[28] Munoz-Bonilla A., Fernández-García M. (2012). Polymeric materials with antimicrobial activity. Prog. Polym. Sci..

[29] Seaman S. (2002). Dressing selection in chronic wound management. J. Am. Podiatr. Med. Assoc..

[30] Prasetyo E.N., Kudanga T., Steiner W., Murkovic M., Nyanhongo G.S., Guebitz G.M. (2009). Antioxidant activity assay based on laccase-generated radicals. Anal. Bioanal. Chem..

[31] Roleira F.M., Tavares-da-Silva E.J., Varela C.L., Costa S.C., Silva T., Garrido J., Borges F. (2015). Plant derived and dietary phenolic antioxidants: Anticancer properties. Food Chem..

[32] Heleno S.A., Martins A., Queiroz M.J.R., Ferreira I.C. (2015). Bioactivity of phenolic acids: Metabolites versus parent compounds: A review. Food Chem..

[33] Fresco P., Borges F., Diniz C., Marques M. (2006). New insights on the anticancer properties of dietary polyphenols. Med. Res. Rev..

[34] Thiem B., Goślińska O. (2004). Antimicrobial activity of rubus chamaemorus leaves. Fitoterapia.

[35] Sakihama Y., Cohen M.F., Grace S.C., Yamasaki H. (2002). Plant phenolic antioxidant and prooxidant activities: Phenolics-induced oxidative damage mediated by metals in plants. Toxicology.

[36] Amaral S., Mira L., Nogueira J., da Silva A.P., Florêncio M.H. (2009). Plant extracts with anti-inflammatory properties—A new approach for characterization of their bioactive compounds and establishment of structure–antioxidant activity relationships. Biorgan. Med. Chem..

[37] Landis S.J. (2008). Chronic wound infection and antimicrobial use. Adv. Skin Wound Care.

[38] Kim H., Kawazoe T., Han D.W., Matsumara K., Suzuki S., Tsutsumi S., Hyon S.H. (2008). Enhanced wound healing by an epigallocatechin gallate-incorporated collagen sponge in diabetic mice. Wound Repair Regen..

[39] Schweigert N., Zehnder A.J., Eggen R.I. (2001). Chemical properties of catechols and their molecular modes of toxic action in cells, from microorganisms to mammals. Environ. Microbiol..

[40] Nagababu E., Rifkind J.M., Boindala S., Nakka L. (2010). Assessment of antioxidant activity of eugenol in vitro and in vivo. Free Radicals and Antioxidant Protocols.

[41] Puertas-Bartolomé M., Fernández-Gutiérrez M., García-Fernández L., Vázquez-Lasa B., San Román J. (2018). Biocompatible and bioadhesive low molecular weight polymers containing long-arm catechol-functionalized methacrylate. Eur. Polym. J..

[42] Huber D., Grzelak A., Baumann M., Borth N., Schleining G., Nyanhongo G.S., Guebitz G.M. (2018). Anti-inflammatory and anti-oxidant properties of laccase-synthesized phenolic-*O*-carboxymethyl chitosan hydrogels. New Biotechnol..

[43] Barclay L.R.C., Edwards C., Vinqvist M.R. (1999). Media effects on antioxidant activities of phenols and catechols. J. Am. Chem. Soc..

[44] Justino G.C., Correia C.F., Mira L., Borges dos Santos R.M., Martinho Simões J.A., Silva A.M., Santos C., Gigante B. (2006). Antioxidant activity of a catechol derived from abietic acid. J. Agric. Food Chem..

[45] Silva M.M., Santos M.R., Caroço G., Rocha R., Justino G., Mira L. (2002). Structure-antioxidant activity relationships of flavonoids: A re-examination. Free Radic. Res..

[46] Heijnen C., Haenen G., Van Acker F., Van der Vijgh W., Bast A. (2001). Flavonoids as peroxynitrite scavengers: The role of the hydroxyl groups. Toxicol. In Vitro.

[47] Heijnen C.G., Haenen G.R., Minou Oostveen R., Stalpers E.M., Bast A. (2002). Protection of flavonoids against lipid peroxidation: The structure activity relationship revisited. Free Radic. Res..

[48] Heijnen C.G., Haenen G.R., Vekemans J.A., Bast A. (2001). Peroxynitrite scavenging of flavonoids: Structure activity relationship. Environ. Toxicol. Pharmacol..

[49] Boots A.W., Haenen G.R., den Hartog G.J., Bast A. (2002). Oxidative damage shifts from lipid peroxidation to thiol arylation by catechol-containing antioxidants. Biochim. Biophys. Acta (BBA) Mol. Cell Biol. Lipids.

[50] Lee H., Dellatore S.M., Miller W.M., Messersmith P.B. (2007). Mussel-inspired surface chemistry for multifunctional coatings. Science.

[51] Lee B.P., Dalsin J.L., Messersmith P.B. (2002). Synthesis and gelation of dopa-modified poly(ethylene glycol) hydrogels. Biomacromolecules.

[52] Scognamiglio F., Travan A., Borgogna M., Donati I., Marsich E., Bosmans J.W.A.M., Perge L., Foulc M.P., Bouvy N.D., Paoletti S. (2016). Enhanced bioadhesivity of dopamine-functionalized polysaccharidic membranes for general surgery applications. Acta Biomater..

[53] Han L., Lu X., Liu K., Wang K., Fang L., Weng L.-T., Zhang H., Tang Y., Ren F., Zhao C. (2017). Mussel-inspired adhesive and tough hydrogel based on nanoclay confined dopamine polymerization. ACS Nano.

[54] GhavamiNejad A., Rajan Unnithan A., Ramachandra Kurup Sasikala A., Samarikhalaj M., Thomas R.G., Jeong Y.Y., Nasseri S., Murugesan P., Wu D., Hee Park C. (2015). Mussel-inspired electrospun nanofibers functionalized with size-controlled silver nanoparticles for wound dressing application. ACS Appl. Mater. Interfaces.

[55] Lu D., Wang H., Li T.E., Li Y., Dou F., Sun S., Guo H., Liao S., Yang Z., Wei Q. (2017). Mussel-inspired thermoresponsive polypeptide–pluronic copolymers for versatile surgical adhesives and hemostasis. ACS Appl. Mater. Interfaces.

[56] Sato T., Aoyagi T., Ebara M., Auzély-Velty R. (2017). Catechol-modified hyaluronic acid: In situ-forming hydrogels by auto-oxidation of catechol or photo-oxidation using visible light. Polym. Bull..

[57] Jeon E.Y., Choi B.-H., Jung D., Hwang B.H., Cha H.J. (2017). Natural healing-inspired collagen-targeting surgical protein glue for accelerated scarless skin regeneration. Biomaterials.

[58] Scognamiglio F., Travan A., Rustighi I., Tarchi P., Palmisano S., Marsich E., Borgogna M., Donati I., de Manzini N., Paoletti S. (2016). Adhesive and sealant interfaces for general surgery applications. J. Biomed. Mater. Res. Part B Appl. Biomater..

[59] Winter G.D. (1962). Formation of the scab and the rate of epithelization of superficial wounds in the skin of the young domestic pig. Nature.

[60] Kim M., Ondrusek B.A., Lee C., Douglas W.G., Chung H. (2018). Synthesis of lightly crosslinked zwitterionic polymer-based bioinspired adhesives for intestinal tissue sealing. J. Polym. Sci. Part A Polym. Chem..

[61] Czuba U., Quintana R., De Pauw-Gillet M.C., Bourguignon M., Moreno-Couranjou M., Alexandre M., Detrembleur C., Choquet P. (2018). Atmospheric plasma deposition of methacrylate layers containing catechol/quinone groups: An alternative to polydopamine bioconjugation for biomedical applications. Adv. Healthc. Mater..

[62] Xu B., Sun X., Wu C., Hu J., Huang X. (2017). Construction of catechol-containing semi-fluorinated asymmetric polymer brush via successive raft polymerization and atrp. Polym. Chem..

[63] Hadjesfandiari N., Weinhart M., Kizhakkedathu J.N., Haag R., Brooks D.E. (2018). Development of antifouling and bactericidal coatings for platelet storage bags using dopamine chemistry. Adv. Healthc. Mater..

[64] Fort R., Polyzoidis T. (1976). Intrinsic viscosity-molecular weight relationships for poly(2-hydroxyethyl methacrylate). Eur. Polym. J..

[65] Xi P., Cheng K., Sun X., Zeng Z., Sun S. (2011). Fluorescent magnetic nanoparticles based on a ruthenium complex and Fe_3_O_4_. J. Mater. Chem..

[66] Paez J.I., Ustahüseyin O., Serrano C., Ton X.-A., Shafiq Z., Auernhammer G.N.K., d’Ischia M., del Campo A.N. (2015). Gauging and tuning cross-linking kinetics of catechol-peg adhesives via catecholamine functionalization. Biomacromolecules.

[67] Wang S.Y., Lan X.Y., Xiao J.H., Yang J.C., Kao Y.T., Chang S.T. (2008). Antiinflammatory activity of lindera erythrocarpa fruits. Phytother. Res..

[68] Guevara I., Iwanejko J., Dembińska-Kieć A., Pankiewicz J., Wanat A., Anna P., Goła̧bek I., Bartuś S., Malczewska-Malec M., Szczudlik A. (1998). Determination of nitrite/nitrate in human biological material by the simple griess reaction. Clin. Chim. Acta.

[69] Choi M.S., Lee S.H., Cho H.S., Kim Y., Yun Y.P., Jung H.Y., Jung J.K., Lee B.C., Pyo H.B., Hong J.T. (2007). Inhibitory effect of obovatol on nitric oxide production and activation of nf-κb/map kinases in lipopolysaccharide-treated raw 264.7 cells. Eur. J. Pharmacol..

[70] Jansen J.F., Houben E.E., Tummers P.H., Wienke D., Hoffmann J. (2004). Real-time infrared determination of photoinitiated copolymerization reactivity ratios: Application of the hilbert transform and critical evaluation of data analysis techniques. Macromolecules.

[71] Aguilar M.R., Gallardo A., Fernández M.D.M., Román J.S. (2002). In situ quantitative 1 h nmr monitoring of monomer consumption: A simple and fast way of estimating reactivity ratios. Macromolecules.

[72] Lopez-Donaire M.L., Sussman E.M., Fernandez-Gutierrez M., Mendez-Vilas A., Ratner B.D., Vazquez-Lasa B., San Roman J. (2012). Amphiphilic self-assembled “polymeric drugs”: Morphology, properties, and biological behavior of nanoparticles. Biomacromolecules.

[73] Lopez Donaire M.L., Parra-Caceres J., Vazquez-Lasa B., Garcia-Alvarez I., Fernandez-Mayoralas A., Lopez-Bravo A., San Roman J. (2009). Polymeric drugs based on bioactive glycosides for the treatment of brain tumours. Biomaterials.

[74] García-Fernández L., Aguilar M.A.R., Fernández M.A.M., Lozano R.M., Giménez G., Román J.S. (2010). Antimitogenic polymer drugs based on amps: Monomer distribution bioactivity relationship of water-soluble macromolecules. Biomacromolecules.

[75] Standardization I.O.F. (2009). Biological Evaluation of Medical Devices—Part 5: Tests for In Vitro Cytotoxicity.

[76] Huang J., de Paulis T., May J.M. (2004). Antioxidant effects of dihydrocaffeic acid in human ea. Hy926 endothelial cells. J. Nutr. Biochem..

[77] Lee B.P., Huang K., Nunalee F.N., Shull K.R., Messersmith P.B. (2004). Synthesis of 3,4-dihydroxyphenylalanine (dopa) containing monomers and their co-polymerization with peg-diacrylate to form hydrogels. J. Biomater. Sci. Polym. Ed..

[78] Larrosa M., Luceri C., Vivoli E., Pagliuca C., Lodovici M., Moneti G., Dolara P. (2009). Polyphenol metabolites from colonic microbiota exert anti-inflammatory activity on different inflammation models. Mol. Nutr. Food Res..

[79] Odian G. (2004). Radical chain polymerization. Principles of Polymerization.

[80] Ahn B.K., Lee D.W., Israelachvili J.N., Waite J.H. (2014). Surface-initiated self-healing of polymers in aqueous media. Nat. Mater..

[81] Glass P., Chung H., Washburn N.R., Sitti M. (2009). Enhanced reversible adhesion of dopamine methacrylamide-coated elastomer microfibrillar structures under wet conditions. Langmuir.

[82] Fernández-Quiroz D., González-Gómez Á., Lizardi-Mendoza J., Vázquez-Lasa B., Goycoolea F.M., San Román J., Argüelles-Monal W.M. (2015). Effect of the molecular architecture on the thermosensitive properties of chitosan-*g*-poly(*N*-vinylcaprolactam). Carbohydr. Polym..

[83] Bölgen N., Aguilar M.R., Fernández M.D.M., Gonzalo-Flores S., Villar-Rodil S., San Román J., Pişkin E. (2015). Thermoresponsive biodegradable hema–lactate–dextran-co-nipa cryogels for controlled release of simvastatin. Artif. Cells Nanomed. Biotechnol..

[84] Rojo L., Barcenilla J.M., Vázquez B., González R., San Román J. (2008). Intrinsically antibacterial materials based on polymeric derivatives of eugenol for biomedical applications. Biomacromolecules.

[85] Shah S., Pal A., Gude R., Devi S. (2010). Synthesis and characterization of thermo-responsive copolymeric nanoparticles of poly(methyl methacrylate-*co*-*N*-vinylcaprolactam). Eur. Polym. J..

[86] Qiu X., Sukhishvili S.A. (2006). Copolymerization of n-vinylcaprolactam and glycidyl methacrylate: Reactivity ratio and composition control. J. Polym. Sci. Part A Polym. Chem..

[87] Gallardo A., Aguilar M.R., Abraham G.A., San Román J. (2004). Chain copolymerization reactions: An algorithm to predict the reaction evolution with conversion. J. Chem. Educ..

[88] Akiyoshi K., Deguchi S., Tajima H., Nishikawa T., Sunamoto J. (1997). Microscopic structure and thermoresponsiveness of a hydrogel nanoparticle by self-assembly of a hydrophobized polysaccharide. Macromolecules.

[89] Li L., Smitthipong W., Zeng H. (2015). Mussel-inspired hydrogels for biomedical and environmental applications. Polym. Chem..

[90] Liu H., Qu X., Kim E., Lei M., Dai K., Tan X., Xu M., Li J., Liu Y., Shi X. (2018). Bio-inspired redox-cycling antimicrobial film for sustained generation of reactive oxygen species. Biomaterials.

[91] Ye M., Jiang R., Zhao J., Zhang J., Yuan X., Yuan X. (2015). In situ formation of adhesive hydrogels based on pl with laterally grafted catechol groups and their bonding efficacy to wet organic substrates. J. Mater. Sci. Mater. Med..

[92] Brubaker C.E., Messersmith P.B. (2011). Enzymatically degradable mussel-inspired adhesive hydrogel. Biomacromolecules.

[93] Cencer M., Murley M., Liu Y., Lee B.P. (2015). Effect of nitro-functionalization on the cross-linking and bioadhesion of biomimetic adhesive moiety. Biomacromolecules.

[94] Xu J., Strandman S., Zhu J.X.X., Barralet J., Cerruti M. (2015). Genipin-crosslinked catechol-chitosan mucoadhesive hydrogels for buccal drug delivery. Biomaterials.

[95] Lee Y., Chung H.J., Yeo S., Ahn C.H., Lee H., Messersmith P.B., Park T.G. (2010). Thermo-sensitive, injectable, and tissue adhesive sol-gel transition hyaluronic acid/pluronic composite hydrogels prepared from bio-inspired catechol-thiol reaction. Soft Matter.

[96] Kim K., Kim K., Ryu J.H., Lee H. (2015). Chitosan-catechol: A polymer with long-lasting mucoadhesive properties. Biomaterials.

[97] Bonakdar S., Emami S.H., Shokrgozar M.A., Farhadi A., Ahmadi S.A.H., Amanzadeh A. (2010). Preparation and characterization of polyvinyl alcohol hydrogels crosslinked by biodegradable polyurethane for tissue engineering of cartilage. Mater. Sci. Eng. C.

[98] Ryu J.H., Lee Y., Do M.J., Jo S.D., Kim J.S., Kim B.S., Im G.I., Park T.G., Lee H. (2014). Chitosan-g-hematin: Enzyme-mimicking polymeric catalyst for adhesive hydrogels. Acta Biomater..

[99] Kalyanaraman B., Premovic P., Sealy R.C. (1987). Semiquinone anion radicals from addition of amino acids, peptides, and proteins to quinones derived from oxidation of catechols and catecholamines. An esr spin stabilization study. J. Biol. Chem..

[100] Alegria A.E., Sanchez-Cruz P., Kumar A., Garcia C., Gonzalez F.A., Orellano A., Zayas B., Gordaliza M. (2008). Thiols oxidation and covalent binding of bsa by cyclolignanic quinones are enhanced by the magnesium cation. Free Radic. Res..

[101] Ryu J.H., Lee Y., Kong W.H., Kim T.G., Park T.G., Lee H. (2011). Catechol-functionalized chitosan/pluronic hydrogels for tissue adhesives and hemostatic materials. Biomacromolecules.

[102] Cencer M., Liu Y., Winter A., Murley M., Meng H., Lee B.P. (2014). Effect of ph on the rate of curing and bioadhesive properties of dopamine functionalized poly(ethylene glycol) hydrogels. Biomacromolecules.

[103] Chen W., Shen X., Hu Y., Xu K., Ran Q., Yu Y., Dai L., Yuan Z., Huang L., Shen T. (2017). Surface functionalization of titanium implants with chitosan-catechol conjugate for suppression of ros-induced cells damage and improvement of osteogenesis. Biomaterials.

[104] Wu M.-S., Sun D.-S., Lin Y.-C., Cheng C.-L., Hung S.-C., Chen P.-K., Yang J.-H., Chang H.-H. (2015). Nanodiamonds protect skin from ultraviolet b-induced damage in mice. J. Nanobiotechnol..

[105] Mencucci R., Mercatelli L., Fusi F., Ponchietti C., Monici M., Menchini U. (2006). Acrysof natural intraocular lens optical characteristics during and after different doses of ultraviolet-visible light illumination. J. Cataract Refract. Surg..

[106] Gantwerker E.A., Hom D.B. (2012). Skin: Histology and physiology of wound healing. Clin. Plast. Surg..

[107] Zhong J., Hu N., Xiong X., Lei Q., Li L. (2011). A novel promising therapy for skin aging: Dermal multipotent stem cells against photoaged skin by activation of TGF-β/Smad and p38 MAPK signaling pathway. Med. Hypotheses.

[108] Xian D., Zhong J., Gao X. (2017). A new approach for skin-derived precursors to ameliorate skin photodamage through activation of nrf2 signaling pathway. Curr. Signal Transduct. Ther..

[109] Pullar J.M., Carr A.C., Vissers M. (2017). The roles of vitamin C in skin health. Nutrients.

[110] Jiang S., Ma B.C., Reinholz J., Li Q., Wang J., Zhang K.A., Landfester K., Crespy D. (2016). Efficient nanofibrous membranes for antibacterial wound dressing and uv protection. ACS Appl. Mater. Interfaces.

[111] Kim T.-S., Cha J.-R., Gong M.-S. (2017). Investigation of the antimicrobial and wound healing properties of silver nanoparticle-loaded cotton prepared using silver carbamate. Text. Res. J..

[112] Rajwade J., Paknikar K., Kumbhar J. (2015). Applications of bacterial cellulose and its composites in biomedicine. Appl. Microbiol. Biotechnol..

[113] Araujo A., de Melo M., Rabelo T., Nunes P., Santos S., Serafini M., Santos M., Quintans-Júnior L., Gelain D. (2015). Review of the biological properties and toxicity of usnic acid. Nat. Prod. Res..

[114] Espada J., Matabuena M., Salazar N., Lucena S., Kourani O., Carrasco E., Calvo M., Rodriguez C., Reyes E., Gonzalez S. (2015). Cryptomphalus aspersa mollusc eggs extract promotes migration and prevents cutaneous ageing in keratinocytes and dermal fibroblasts in vitro. Int. J. Cosmet. Sci..

[115] Park K.-M., Yoo J.-H., Shin Y.-J. (2012). Effects of egg shell membrane hydrolysates on skin whitening, wound healing, and uv-protection. Korean J. Food Sci. Anim. Resour..

[116] Sohn M., Malburet C., Baptiste L., Prigl Y. (2017). Development of a synthetic substrate for the in vitro performance testing of sunscreens. Skin Pharmacol. Physiol..

[117] Osterwalder U., Sohn M., Herzog B. (2014). Global state of sunscreens. Photodermatol. Photoimmunol. Photomed..

[118] Mavon A., Zahouani H., Redoules D., Agache P., Gall Y., Humbert P. (1997). Sebum and stratum corneum lipids increase human skin surface free energy as determined from contact angle measurements: A study on two anatomical sites. Colloids Surf. B Biointerfaces.

[119] Ginn M., Noyes C., Jungermann E. (1968). The contact angle of water on viable human skin. J. Colloid Interface Sci..

[120] Francesko A., da Costa D.S., Lisboa P., Reis R.L., Pashkuleva I., Tzanov T. (2012). Gags-thiolated chitosan assemblies for chronic wounds treatment: Control of enzyme activity and cell attachment. J. Mater. Chem..

[121] Perelshtein I., Ruderman E., Francesko A., Fernandes M.M., Tzanov T., Gedanken A. (2014). Tannic acid nps–synthesis and immobilization onto a solid surface in a one-step process and their antibacterial and anti-inflammatory properties. Ultrason. Sonochem..

[122] Cai Y., Luo Q., Sun M., Corke H. (2004). Antioxidant activity and phenolic compounds of 112 traditional chinese medicinal plants associated with anticancer. Life Sci..

[123] Wang H., Joseph J.A. (1999). Quantifying cellular oxidative stress by dichlorofluorescein assay using microplate reader1. Free Radic. Biol. Med..

[124] Li Y.F., Zhang H.T., Xin L. (2018). Hyaluronic acid-modified polyamidoamine dendrimer g5-entrapped gold nanoparticles delivering metase gene inhibits gastric tumor growth via targeting cd44+ gastric cancer cells. J. Cancer Res. Clin. Oncol..

[125] Dileep K.V., Tintu I., Mandal P.K., Karthe P., Haridas M., Sadasivan C. (2012). Binding to pla2 may contribute to the anti-inflammatory activity of catechol. Chem. Biol. Drug Des..

[126] Zheng L.T., Ryu G.-M., Kwon B.-M., Lee W.-H., Suk K. (2008). Anti-inflammatory effects of catechols in lipopolysaccharide-stimulated microglia cells: Inhibition of microglial neurotoxicity. Eur. J. Pharmacol..

[127] Kojima M., Morisaki T., Izuhara K., Uchiyama A., Matsunari Y., Katano M., Tanaka M. (2000). Lipopolysaccharide increases cyclo-oxygenase-2 expression in a colon carcinoma cell line through nuclear factor-κb activation. Oncogene.

