# Supplementary material for: Bioactive and Bioadhesive Catechol Conjugated Polymers for Tissue Regeneration
Source: Polymers (Basel). 2018 Jul 13;10(7):768. doi: 10.3390/polym10070768 (PMC6403640; doi:10.3390/polym10070768)
Supplement: Supplementary file 1 [file polymers-10-00768-s001.pdf]

Supplementary Materials

Bioactive and Bioadhesive Catechol Conjugated Polymers for Tissue Regeneration

María Puertas-Bartolomé [1,2], Blanca Vázquez-Lasa [1,2,*] and Julio San Román [1,2]

1 Institute of Polymer Science and Technology, ICTP-CSIC, Juan de la Cierva 3, 28006 Madrid, Spain; mpuertas@ictp.csic.es (M.P.-B.); jsroman@ictp.csic.es (J.S.R.)
2 CIBER's Bioengineering, Biomaterials and Nanomedicine, CIBER-BBN, Health Institute Carlos III, C/Monforte de Lemos 3-5, Pabellón 11, 28029 Madrid, Spain
* Correspondence: bvazquez@ictp.csic.es; Tel.: +34-915-618-806 (ext. 921522)

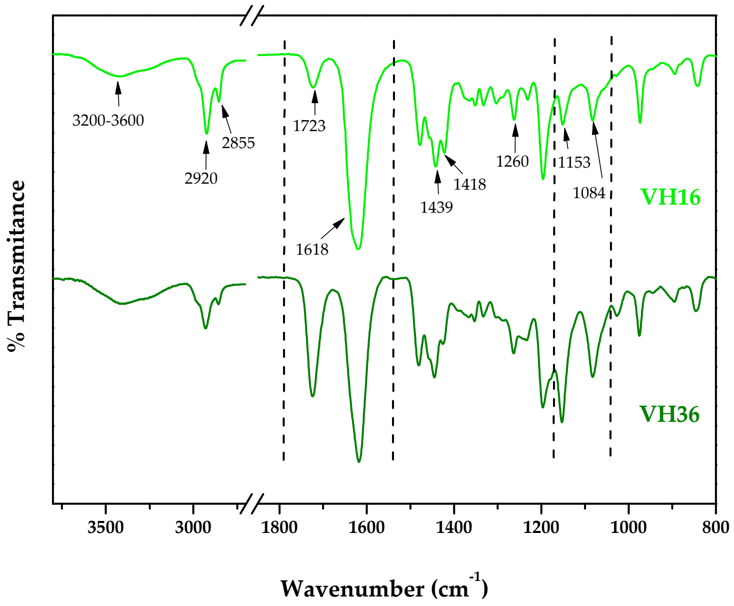

Figure S1. FTIR spectra of VH copolymers.

Table S1. Wavenumber and corresponding assignments for the main peaks in FTIR spectra of VH copolymers.

Assignment.	Wavenumber (cm^{-1})
υO-H H-bonded	3200-3600
υ C-H in CH_2 and CH_3 groups (asymmetric and symmetric)	2920
	2855
υ C=O in ester group	1723
υ C=O amide I	1618
δ C-H in CH_2 and CH_3 groups (asymmetrical)	1439
δ -CH_3 (symmetrical)	1418
Twisting and wagging CH_2	1260
υ C-O asym.	1153
υ C-O sym.	1084

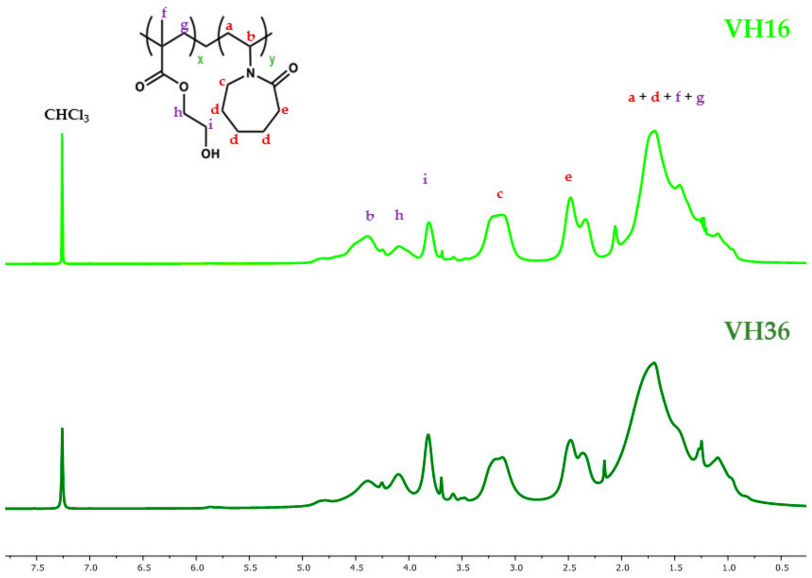

Figure S2. ^{1}H-NMR spectra of VH copolymers in CDCl3.

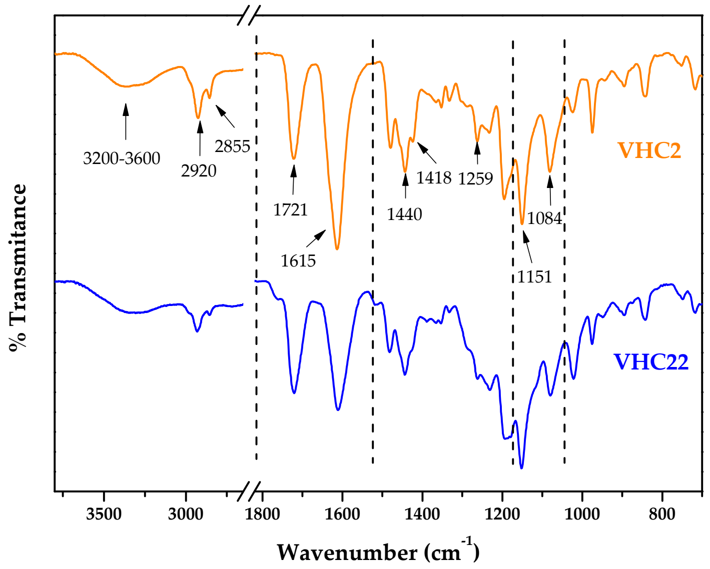

Figure S3. FTIR spectra of VHC terpolymers.

Table S2. Wavenumber and corresponding assignments for the main peaks in FTIR spectra of VHC terpolymers.

Assignment	Wavenumber (cm^{-1})
υO-H H-bonded	3200-3600
υ C-H in $CH_2$and CH_3groups (asymmetric and symmetric)	2925<br>2855
υ C=O in ester group	1721
υ C=O amide I	1615
δ C-H in $CH_2$and CH_3groups (asymmetrical)	1439
δ -CH_3(symmetrical)	1418
Twisting and wagging CH_2	1259
υ C-O asym.	1151
υ C-O sym.	1084

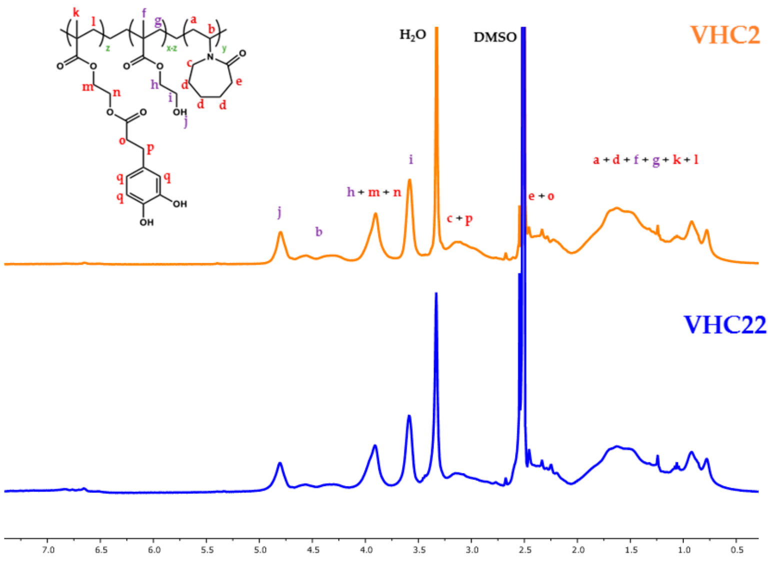

Figure S4. ^{1}H-NMR spectra of VHC terpolymers in DMSO-d_6.
